# A Rare Case of Small Bowel Obstruction in a Patient with Endosalpingiosis, Fitz-Hugh-Curtis Syndrome, and *Chlamydia trachomatis* Pelvic Inflammatory Disease

**DOI:** 10.1155/2022/2451428

**Published:** 2022-10-22

**Authors:** Zhi Kiat Sia, Jodie Trautman, Takako Eva Yabe, James Wykes

**Affiliations:** The Wollongong Hospital, Loftus Street, Wollongong, NSW 2500, Australia

## Abstract

A 19-year-old female has multiple presentations to emergency department with recurrent abdominal pain. During her third presentation, the radiological features were suggestive of high-grade small bowel obstruction in a virgin abdomen. A diagnostic laparoscopy has been performed. The intraoperative findings include a band adhesion between omentum and small bowel mesentery, and perihepatic adhesions consistent with Fitz-Hugh-Curtis syndrome. The histopathology from a biopsy of the macular lesions of the abdominal wall showed endosalpingiosis. The postoperative high vaginal swab was positive for *Chlamydia trachomatis*. The underlying cause of her small bowel obstruction could be due to pelvic inflammatory disease, Fitz-Hugh-Curtis syndrome, or endosalpingiosis. We aimed to create awareness amongst readers that small bowel obstruction in young female patients with no prior abdominal surgery is possible and often difficult to diagnose immediately.

## 1. Introduction

A small bowel obstruction in a virgin abdomen is rare and has to be treated with caution. The potential underlying causes include but not limited to internal hernia, congenital adhesions, endometriosis, and pelvic inflammatory disease. In this case report, we will discuss about the presentation of a young female patient to the hospital, the investigations involved and the management plans. We will also discuss in detailed about Fitz-Hugh-Curtis syndrome, endosalpingiosis, and pelvic inflammatory disease in the context of small bowel obstruction.

## 2. Case Report

In this report, we describe a case of a 19-year-old female who presented to a regional hospital emergency department with increasing colicky abdominal pain, describing at least seven days of obstipation, intolerance to oral intake, and multiple large amounts of vomits. This was her third presentation within nine days. On this occasion, the general surgery service was consulted, and the computed tomography of the abdomen and pelvis with intravenous contrast was performed. Imaging demonstrated a high-grade small bowel obstruction (SBO) with a transition point in the pelvis.

The patient's medical history included primary dysmenorrhea. She tried oral contraceptive pills and Implanon but tolerated them poorly to their effect on mood. A recent gynaecological endocrine panel was unremarkable. She was on the waitlist for a diagnostic laparoscopy to investigate for suspected endometriosis. The previous sexually transmitted infection (STI) screening was reported to be negative. She had not had any abdominal operations and did not take any regular medications.

**Figure 1:**
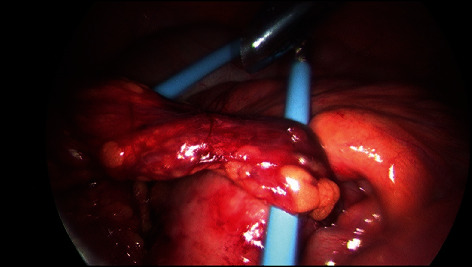
Band of omentum adhered to small bowel mesentery causing SBO.

**Figure 2:**
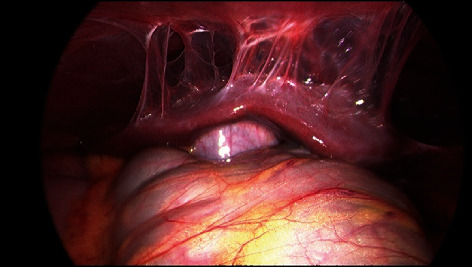
Perihepatic adhesions above the left and right lobes of the liver, consistent with Fitz-Hugh-Curtis syndrome.

**Figure 3:**
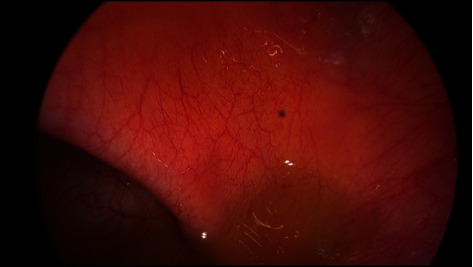
Dark macular lesion on the right abdominal wall.

After gastric decompression and intravenous fluid resuscitation, the patient was transferred to a tertiary referral hospital and proceeded to diagnostic laparoscopy. Operative findings were of dilated loops of small bowel with a band adhesion caused by the omentum adhered to small bowel mesentery (Figure 1). There were also nonobstructing inflammatory adhesions between small bowel loops in the pelvis and perihepatic adhesions involving the left and right lobes of the liver (Figure 2). This was consistent with Fitz-Hugh-Curtis syndrome. Additionally, there were macular lesions on the right abdominal wall and pelvic sidewall (Figure 3) and mixed dark and clear vesicular lesions on the small bowel mesentery. The omental adhesion was divided with diathermy. The small bowel was inspected from duodenojejunal flexure to terminal ileum, demonstrating viable decompressed bowel and no other pathology.

Postoperative gynaecological review elucidated the further history of deep dyspareunia. The high vaginal swab was positive for *Chlamydia trachomatis*. Unexpectedly, the intraoperative histopathology did not identify endometriosis but rather endosalpingiosis. It is a less well-studied entity and has not been associated with intestinal obstruction in published literature. The patient progressed to a complete diet and was discharged on the second postoperative day with gynaecological follow up.

## 3. Discussion

The small bowel obstruction (SBO) in a virgin abdomen is rare and may be caused by congenital adhesions, small bowel tumours, internal hernia, or gallstone ileus [1]. In this case, alternate causes of abdominal pain were initially suspected. Based on the clinical history and the intraoperative lesions identified, endometriosis was initially suspected to be the cause of SBO in this patient [2].

Endosalpingiosis is described as ectopic glands of fallopian tube-type ciliated epithelium. It may occur in female reproductive and other visceral or retroperitoneal organs, either in isolation or concurrently with endometriosis or endocervicosis. The clinical features of endosalpingiosis are not well described. It may be associated with pelvic pain, infertility, and urinary symptoms. Literature reports a histologic relationship between endosalpingiosis and pelvic serous neoplasms, whether as a risk factor or disease process is unclear [3]. Histologically, it is not known to be associated with a localised inflammatory response. For this reason, endosalpingiosis is less likely to be the cause of the adhesive SBO in our patients.

The other diagnosed concurrent pathology was *Chlamydia trachomatis* with pelvic inflammatory adhesions and Fitz-Hugh-Curtis syndrome. The numerous hospital presentations with right upper quadrant pain may have been related to the liver capsule inflammation. SBO caused by PID is rare, and there are only 13 cases reported in the English literature to our knowledge (Table 1). The majority of these had laboratory evidence of *Chlamydia* infection, whilst two reported cases were resolved with conservative management, and the remaining 11 cases required surgical intervention. Five of the reported cases demonstrated SBO associated with tubo-ovarian abscess. Three were SBO due to perihepatic adhesions, and the remaining five were related to peritoneal adhesion. The case described by Haumann et al. [4] bears the most similarity to the reported case. The patient presented in their report had SBO and a history of recent acute *Chlamydial* infection. She also had a band adhesion caused by omentum with an inflammatory attachment to the small bowel. The intraoperative appearance showed only focal area of inflammation with no sclerosis or membrane formation, which is different to sclerosing encapsulating peritonitis (SEP) described in the literature [5].

This is the first reported case of SBO with histological findings of endosalpingiosis. It is however difficult to prove if it was the true causative aetiology for her presentation. Another possibility is that her SBO was due to endometriosis [6–8].. Whilst it was not demonstrated on our patient's biopsies, endometriosis is known to coexist with endosalpingiosis. In addition, this case could be caused by PID-related adhesions, which adds to the sparse literature detailing such cases [9–11].

## 4. Conclusion

In this case report, we have identified a young, fertile female patient with recurrent presentations to the hospital with abdominal pain. She had an underlying SBO with no prior abdominal surgery. The cause of her SBO could be related to the underlying clinical pathologies as discussed above. PID and endometriosis may be important to be identified early to prevent future SBO.

## Figures and Tables

**Table 1:** History, findings, and management of small bowel obstruction in patients with PID.

Authors	Age	History	Management & findings
Baumgardner & McCanse [12]	24	(i) 3-week history of intermittent epigastric pain	(i) Exploratory laparotomy and adhesiolysis (ii) Fibrinous adhesion (iii) PID treated with tetracycline
Harel and Lambrianides [10]	19	(i) Bile-stained emesis (ii) CT abdomen showed a high-grade partial SBO	(i) Conservative management (ii) Antibiotics (iii) Nasogastric tube (iv) Resolution of SBO within 2 days
Haumann et al. [4]	27	(i) Clinical obstructive symptoms (ii) CT abdomen showed an acute SBO with no obvious aetiology	(i) Exploratory laparoscopy and adhesiolysis (ii) The transition point was in the jejunum due to adhesion (iii) Oral antibiotics for 3 weeks to treat Chlamydia trachomatis
Rossi et al. [13]	54	(i) Seven-day history of intermittent obstructive symptoms in a virgin abdomen (ii) X-ray abdomen showed a dilated loop of small bowel anterior to the liver	(i) Exploratory laparotomy and adhesiolysis (ii) Several adhesions between the liver and diaphragm with a loop of jejunum that had herniated between two of these adhesions
Rossi et al. [13]	58	(i) Three-day history of vomiting and crampy abdominal pain (ii) X-ray abdomen showed evidence of complete SBO	(i) Exploratory laparotomy and adhesiolysis (ii) A loop of midileum was entrapped between the adhesions above the right lobe of the liver causing complete obstruction
Pines et al. [14]	35	(i) Abdominal pain and recurrent bile-stained emesis (ii) X-ray abdomen revealed slight dilated small bowel loops with air-fluid levels	(i) Exploratory laparoscopy then low-midline laparotomy (ii) Left and right tubo-ovarian abscess adhered to small bowel identified (iii) A left and right salpingectomy
Martin-Lagos Maldonado et al. [15]	24	(i) Two-day history of vomiting, hypogastric and right iliac fossa pain, fever, and vaginal discharge (ii) Abdominal X-ray showed generalised dilation of small bowel. Pelvic and transvaginal ultrasound showed a heterogeneous cystic lesion on the right ovary and free fluid in the pouch of Douglas	(i) Exploratory laparoscopy then laparotomy. Right salpingectomy, adhesiolysis, and ileal resection (ii) Formation of fibrotic adhesions towards the ileum intestinal wall. Salpingitis with a large right tubo-ovarian abscess (iii) IV ertapenem then oral amoxicillin-clavulanic and metronidazole
Pegg and Owen [16]	18	(i) Recurrent abdominal pain and vomiting (ii) Clinical and plain radiological signs of gastrointestinal obstruction	(i) Exploratory laparotomy, appendicectomy, adhesiolysis, and peritoneal lavage (ii) Peritoneal cavity contained 900 ml of straw-coloured fluid, widespread adhesions between adjacent loops of small bowel
Harris and Lambrianides [11]	18	(i) Two-day history of colicky abdominal pain, bile-stained emesis, and constipation (ii) CT abdomen showed dilated loops of small bowel and a small amount of free fluid within the pelvis	(i) Laparotomy and adhesiolysis (ii) Multiple adhesions at terminal ileum (iii) Free peritoneal fluid and bilateral pyosalpinx (iv) Antibiotics
Ahmed et al. [9]	38	(i) One-day history of severe abdominal pain and three-week history of constipation (ii) CT abdomen and MRI-pelvic showed dilatation of the colon with transition point in the sigmoid colon. Multiple bilateral cystic lesions suspicious for abscesses	(i) Conservative management (ii) Intrauterine device removed (iii) Doxycycline and ceftriaxone
Francesco et al. [17]	49	(i) One-day history of acute abdominal pain, febrile 38.5°C (ii) Plain X-ray was normal (iii) CT abdomen showed dilated loops of proximal small bowel with wall thickening and an air-fluid level	(i) Exploratory laparoscopy and peritoneal lavage (ii) Nonmalodorous pus in the pelvis with signs of peritonitis. The uterus was oedematous, erythematosus, and swollen (iii) Azithromycin and metronidazole
Al-Ghassab et al. [18]	32	(i) One-day history of obstructive symptoms (ii) Abdominal X-ray showed subacute SBO (iii) CT abdomen showed dilated loops of small bowel and left hydrosalpinx	(i) Exploratory laparoscopy and adhesiolysis (ii) Multiple small bowel adhesions at the ileum and between the liver and anterior abdominal wall, free peritoneal fluid, and left hydrosalpinx (iii) Antibiotics
Duffy et al. [19]	23	(i) 12-hour history of abdominal pain with a background history of PID (ii) Normal erect and supine X-ray of the abdomen	(i) Diagnostic laparoscopy then laparotomy and adhesiolysis (ii) Serous fluid in the pouch of Douglas, widespread adhesions on the capsule of the liver, jejunum, and proximal ileum and its mesentery with multiple hard lymph nodes and inflamed omentum
